# Evaluation of the anti-inflammatory, analgesic, anti-pyretic and anti-ulcerogenic potentials of synthetic indole derivatives

**DOI:** 10.1038/s41598-023-35640-4

**Published:** 2023-05-27

**Authors:** Saira Siddique, Khawaja Raees Ahmad, Syed Kashif Nawaz, Abdul Rauf Raza, Syeda Nadia Ahmad, Rabiyah Ali, Iram Inayat, Sadia Suleman, Muhammad Ali Kanwal, Muhammad Usman

**Affiliations:** 1 Department of Zoology, University of Sargodha, Sargodha, Punjab Pakistan; 2 Govt. Graduate Ambala Muslim College, Sargodha, Pakistan; 3 Institute of Chemistry, University of Sargodha, Punjab, Pakistan; 4 University of Chakwal, Chakwal, Pakistan

**Keywords:** Drug discovery, Gastroenterology

## Abstract

A large number of new synthetic compounds are synthesized in the field of heterocyclic chemistry having a variety of biological potentials. In the present study, some synthetic indole derivatives are used to check anti-inflammatory, analgesic, antipyretic and gastroprotective activity in albino mice. Albino mice of either sex of reproductive age were used for each study (n = 5). In anti-inflammatory activity, the negative control (NC) and positive control group animals were treated with normal saline and 10 mg/kg of indomethacin respectively. The treated groups received the twenty four different synthetic chemicals, after 30 min of sub cutaneous injection of carrageenan. In analgesic activity, hot-plate method is used and for each group the latency period was recorded at zero moment of the provision of required dose and after 30, 60, 90, 120 and 180 min. In anti-pyretic activity, Pyrexia was induced by using Brewer's yeast method. Before any treatment and then after duration of 18 h, the rectal temperatures were recorded. Among all the chemicals, only those chemicals which show any potential related to above mentioned activities were selected for gastroprotective activity. The gastroprotective activity was performed to check the gastric ulcers by using 300 mg/kg of single oral dose of indomethacin to animals of all groups except NC group. This study helped to screen out the most potent indole derivatives 3a-II and 4a-II from the 24 synthetic indole derivatives which demonstrated the best biological potential (anti-inflammatory, analgesic, antipyretic, and gastroprotection) as compared to the remaining ones. The micrometric and biochemical results also support the histological findings. Out of the twenty-four novel indole amines tested, 3a-II and 4a-II have shown the effective pharmacological capacity and additionally have not shown any overt and systemic toxicity. Thus these two indole amines need further in-depth pharmacokinetic and pharmacodynamics studies before they are recommended for any pre-clinical trial.

## Introduction

With the increase in human population, the need for higher yield and production of crops is mandatory for meeting the food requirements. For this purpose, several techniques have been developed. One strategy is based on the control of losses associated with insects and pests. The use of poisonous chemicals is introduced in the field of agriculture for the control of insects and pests. The excessive application of insecticides, pesticides, and many other commercially produced chemicals is in practice now for the higher production of crops. These harmful chemicals ultimately enter the food chain and cause multiple physiological and anatomical aberrations in the dependent living organisms^1,2^. The malignancies and the inflammatory reactions are the general outcomes of the toxicity of insecticides and pesticides. Non-steroidal anti-inflammatory drugs (NSAIDs) are the most extensively used analgesic and anti-inflammatory drugs but these are also the major causative agent that not only induces gastric damage but via various mechanisms, also delay the process of healing^3^. The topic of detoxification of these chemicals remained the topic of interest for the avoidance of such health damage.

The indoles are present naturally in some plants and fungi. These are among the most versatile and widely utilized nitrogen-based heterocyclic scaffolds. It has four reactive sites which include the nitrogen atom at position 1, a carbon atom at 3, a sigma bond between C2-N, and the π-bond of the C2-C3 position, also can accept protons from strong acids like HCl and the carbon atom of 3rd position is more easily protonated as compared to the nitrogen atom^4^.pharmacological properties, indole-based compounds are extremely important among heterocyclic structures^4^. Oxypertine, an indole derivative, is an antipsychotic and antidepressant, commonly used to treat schizophrenia^5^. Indole 3-carbinol is a major bioactive component found in cruciferous vegetables. It has been studied for its potential to prevent a variety of malignancies (breast, prostate, colorectal, lymphoma, and trans-placental cancer in offspring)^6–8^. Considering their medicinal potential, the present study will aim to explore the protective roles of novel synthetic indole derivatives against Indo-induced gastric injury. We propose that with their antipyretic, analgesic, and anti-inflammatory properties, these compounds would be gastro-protective against indomethacin-induced gastric ulcerations.
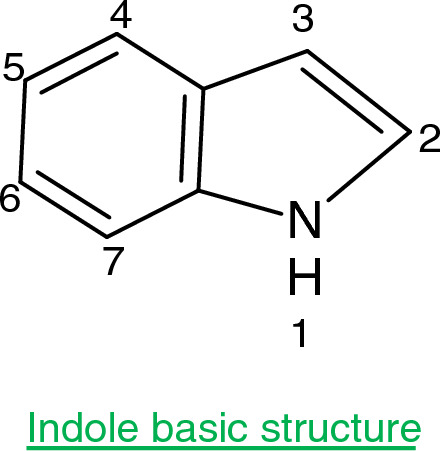


## Methodology

The study was carried out in compliance with ARRIVE guidelines.

### Experimental animals and their maintenance

Adult virgin albino mice of either sex weighing 30–32 g of around 10–12 weeks of age were used in experimental work. These animals were kept in separate iron cages gauzed with stainless steel in the animal house of the Department of Zoology, University of Sargodha under standard housing conditions. All experimental procedures complied with the National Institute of Health Guide for the Care and Use of Laboratory Animals (USA) and were approved by the Ethical review committee of the Department of Zoology, University of Sargodha.

### Dose regime and safety profile

All 24 chemicals were tested at 10 mg/kg concentrations to record analgesic, antipyretic, or anti-inflammatory capacity through intra-gastric exposure. However, none of these chemicals have shown any sign of overt toxicity (frequent optic or oro-anal discharge, profuse urination, lethargy, abstained feeding and improper gait, etc.) or behavioral changes (aggression, restlessness, etc.) on 10 mg/kg or lower intra-gastric exposures.

### Carrageenan-induced paw edema

#### Methodology

All the test chemicals were administrated in a dose of 10 mg/kg to the animals. After 30 min, the 0.01 ml of 1% freshly prepared carrageenan solution was injected subcutaneously into the sub plantar region of the right hind paw. The negative control (NC) and positive control (PC) group animals were treated with normal saline and 10 mg/kg of indomethacin respectively in place of test chemicals. The time taken for the paw edema to subside was recorded using a vernier caliper at different time intervals (20 min, 40 min, 60 min….0.200 min)^9,10^.

### Antipyretic activity

#### Brewer's yeast induced Pyrexia

Antipyretic activities of all chemicals were determined by injecting 20 mg/kg of Brewer's yeast (20% suspension) following the method of Tesema and Makonnen^11^. Animals showing an increase in rectal temperature from 0.3 to 0.6 °C were used for further investigation. These selected animals (n: 5) were distributed in 26 groups. Twenty-four groups received one of the 24 chemicals to be tested (10 mg/kg) while one of the remaining two groups received Paracetamol (150 mg/kg) and the second was given vehicle (1% aqueous DMSO) only. The rectal temperature was recorded by K-type thermocouple for 3 consecutive hours on an hourly basis after respective dose administration with the help of a digital thermocouple (K-type).

#### Analgesic activity (Hot plate method)

Evaluation of the analgesic capacity of the novel chemicals was performed using the temperature tolerance ability of the animals in each experimental group by placing them one by one in a restrainer on the hot plate maintained at 55 °C. The time taken for the first reaction to the heat by each animal was recorded. Individual sensitivity responses include jumping, withdrawal, licking of the paws, etc. The period (latency period) between the moments an animal first placed in the restrainer and the moment it showed any of the above response ware recorded by the digital stopwatch. For each group, the latency period was recorded at zero moments of the provision of the required dose and after 30, 60, 90, 120, and 180 min. These data obtained in treated groups were compared statistically with standard drug (indomethacin 10 mg/kg) and NC (1% aqueous DMSO) group for the significance difference^12,13^.

### Data analysis and statistical applications

Data obtained was analyzed through SPSS.20 software, ANOVA and Tukey’s Multiple Range Test.

### Further investigation

There are twenty four synthetic indole derivatives which were used in this study to check their anti-inflammatory, analgesic and anti-pyretic potential by using different appropriate methods for each activity in albino mice. From this list of chemicals, majority of derivatives have shown null potential in all of three activities, 2 chemicals 389(*N*-(4′-Methoxyphenyl) (4,6-dimethoxy-2,3-diphenyl-1*H*-indol-7-yl)methanimine) and 392(*N*-(3′-Nitrophenyl) (4,6-dimethoxy-2,3-diphenyl-1*H*-indol-7-yl)methanimine) have shown pyretic, anti-analgesic and inflammatory effects and only two chemicals 3a-II(*N*-(4′-Chlorophenyl) (4,6-dimethoxy-2,3-diphenyl-1*H*-indol-7-yl)methanimine) and 4a-II(*N*-(4′-Methylphenyl) (4,6-dimethoxy-2,3-diphenyl-1*H*-indol-7-yl)methanimine)have given the desired effects like anti-inflammatory, analgesic and anti-pyretic and were chosen for further investigation.

For in-depth gastro-protective screening of the 3a-II and 4a-II seventy adult albino mice of either sex were used in further experimental work. These animals were kept in 7 groups (10 in each) in stainless steel meshed iron cages.

### Gastroprotective activity

#### Drugs and chemicals

The experimental dilutions (20 mg/kg and 40 mg/kg) of 3a-II and 4a-II chemicals were prepared by first dissolving the appropriate amount in DMSO and then diluting with normal saline to achieve the required strength. Indomethacin was also dissolved in 0.9% saline to get the desired dose of 300 mg/kg.

#### Indomethacin-induced gastric ulcer in Albino mice

In the available literature, different concentrations have been used to induce peptic ulcer for example 18 mg/kg^14^, and 80 mg/kg^15^. Similar initial trials were carried out to determine the effective oral dose to induce gastric ulceration in mice in this study. Based on the results 300 mg/kg single oral dose of indomethacin was selected for further study.

### Animal groups

Nine experimental groups and their dose profile was as per detailed below

1. *Negative control (NC) group*: received 0.1 ml normal saline (NS) and after 30 min received 0.1 ml normal saline containing 1%DMSO (NSD).
2. *Positive control (PC) group:* received 300 mg/kg of indomethacin and after 30 min 0.1 ml NSD.
3. *PC-O (Omeprazole) group:* received 20 mg/kg omeprazole after 30 min of administration of 300 mg/kg of indomethacin^16^,
4. *3a-II (20 mg//kg) group*: received 20 mg/kg 3a-II after 30 min of administration of 300 mg/kg of indomethacin.
5. *3a-II (40 mg/kg) group*: received 40 mg/kg 3a-II after 30 min of administration of 300 mg/kg of indomethacin.
6. *4a-II (20 mg/kg) group*: received 20 mg/kg 4a-II after 30 min of administration of 300 mg/kg of indomethacin.
7. *4a-II (40 mg/kg) group*: received 40 mg/kg 4a-II after 30 min of administration of 300 mg/kg of indomethacin.
8. *3a-4a-II (10 mg/kg) group*: received mixture containing 10 mg/kg of 3a-II and 40 mg/kg of 4a-II after 30 min of administration of 300 mg/kg of indomethacin.
9. *3a-4a-II (20 mg/kg) group*: received mixture containing 20 mg/kg of 3a-II and 40 mg/kg of 4a-II after 30 min of administration of 300 mg/kg of indomethacin.

The dose administered volume in each case remained 0.1 ml (containing the required strength of the active ingredients). After two hours of drug administration, all mice were euthanized through cervical dislocation and the visceral chamber was opened through a mid-abdominal incision to record any signs of toxicity in situ.

### Histological processing

Then stomachs were excised for gastro-protective studies with the help of forceps and scissors. Each stomach was cut opened through a longitudinal cut along the middle of the greater curvature, washed with normal saline to remove gastric contents to find out the intensity and spread of gastric ulcer along the minor curvature under a glass magnifier (3×) and dissecting binoculars (10×). The gastric lesions were also recorded as digital photographs using a 7.2 MP “Sony DS –W35 camera in super macro mode (3× optical magnifications). After the estimation of gastric lesions, a small highly inflamed portion of the corpus region of the stomach wall was taken from the indomethacin group followed by taking the same glandular portion from all groups for fixation in alcoholic formaldehyde solution for 24 h. After fixation, tissues were dehydrated in 50%, 70%, 90%, and absolute ethanol followed by wax embedding in molten paraffin wax. Then wax tissues were sectioned serially (2–3 µ thick) on a rotary microtome. The serial sections were finally affixed on the albumenized glass slides for hematoxylin and eosin staining for microscopic studies and digital photomicrography^17^.

### Photography and micrometric studies

Digital snapshots of selected sections of tissues were captured in a Sony (DSC-W35) 7.2 MP digital camera attached to a trinocular research microscope (Labomed CXR2) with 400× magnifications. Measurements were taken from twenty randomly selected areas from each section using digital scales in corelDRAW11®. The micrometric data obtained was used to calculate group means ± SEM values. Thus measurements were made for mean cross-sectional area by using the following formula:

$$ {\text{CSA}} = ({\text{length}} \times {\text{width}}/{4})\,\pi $$

### Blood sampling and testing

Blood sample from each animal was obtained directly from the beating heart with help of a 3 ml syringe (left and right ventricles in succession). On average 1 ml of blood can easily be recovered by employing this beating heart recovery technique. The blood samples were further processed for hematological tests.

### Data analysis and statistical applications

Data obtained were analyzed through SPSS.20 software and were expressed as mean ± SEM. The significance of the difference between mean values was determined by using one way ANOVA and Tukey’s Multiple Range Test. The values showed p˂0.05 were considered significant^17,18^.

### Ethics approval

The study protocol was approved by the Ethical committee of the Department of Zoology, University of Sargodha (Reference Number UOS/DS/293, Dated 23-2-2022). All experimental procedures complied with the National Institute of Health Guide for the Care and Use of Laboratory Animals (USA).

### Consent to participate

All the methods in the present study were carried out under relevant guidelines and regulations.

## Results

See Table 1.

**Table 1:** General responses to the anti-inflammatory, analgesic and anti-pyretic activities of the individual chemicals are enumerated in the table below.

S. no	Code	IUPAC Names of the Compounds	Anti-inflammatory activity	Analgesic activity	Anti-pyretic activity
1	349	4,6-Dimethoxy-2,3-diphenyl-1*H*-indole	×	×	×
2	364	4,6-Dimethoxy-2,3-diphenyl-1*H*-indole-7-carbaldehyde	×	×	×
3	382	*N*-Phenyl (4,6-dimethoxy-2,3-diphenyl-1*H*-indol-7-yl)methanimine	×	×	×
4	384	*N*-(3′-Chlorophenyl) (4,6-dimethoxy-2,3-diphenyl-1*H*-indol-7-yl)methanimine	×	×	×
5	4a-ll	*N*-(4′-Methylphenyl) (4,6-dimethoxy-2,3-diphenyl-1*H*-indol-7-yl)methanimine	ǂǂ	Aǂǂ	Pǂǂ
6	385	*N*-(2′,3′-Dichlorophenyl) (4,6-dimethoxy-2,3-diphenyl-1*H*-indol-7-yl)methanimine	×	×	×
7	3a-ll	*N*-(4′-Chlorophenyl) (4,6-dimethoxy-2,3-diphenyl-1*H*-indol-7-yl)methanimine	ǂǂ	Aǂǂ	Pǂǂ
8	387	*N*-(3′-Methylphenyl) (4,6-dimethoxy-2,3-diphenyl-1*H*-indol-7-yl)methanimine	×	×	×
9	388	*N*-(2′,3′-Dimethylphenyl) (4,6-dimethoxy-2,3-diphenyl-1*H*-indol-7-yl)methanimine	×	×	×
10	389	*N*-(4′-Methoxyphenyl) (4,6-dimethoxy-2,3-diphenyl-1*H*-indol-7-yl)methanimine	¥	¶	Ð
11	390	*N*-(3′,4′,5′-Trimethoxyphenyl) (4,6-dimethoxy-2,3-diphenyl-1*H*-indol-7-yl)methanimine	×	×	×
12	392	*N*-(3′-Nitrophenyl) (4,6-dimethoxy-2,3-diphenyl-1*H*-indol-7-yl)methanimine	¥	¶	Ð
13	21-a	4,5,6-Trimethoxy-2,3-diphenyl-7-phenylaminomethyl-1*H*-indole	×	×	×
14	21-b	4,5,6-Trimethoxy-7-(3′-methylphenylaminomethyl)-2,3-diphenyl-1*H*-indole	×	×	×
15	21-c	4,5,6-Trimethoxy-7-(4′-methylphenylaminomethyl)-2,3-diphenyl-1*H*-indole	×	×	×
16	21-d	4,5,6-Trimethoxy-7-(4′-methoxyphenylaminomethyl)-2,3-diphenyl-1*H*-indole	×	×	×
17	21-e	7-(3′-Chlorophenylaminomethyl)-4,5,6-trimethoxy-2,3-diphenyl-1*H*-indole	×	×	×
18	21-f	7-(4′-Chlorophenylaminomethyl)-4,5,6-trimethoxy-2,3-diphenyl-1*H*-indole	×	×	×
19	21-g	7-(3′,4′-Dichlorophenylaminomethyl)-4,5,6-trimethoxy-2,3-diphenyl-1*H*-indole	×	×	×
20	21-h	4,5,6-Trimethoxy-7-(3′-nitrophenylaminomethyl)-2,3-diphenyl-1*H*-indole	×	×	×
21	21-i	4,5,6-Trimethoxy-7-(4′-nitrophenylaminomethyl)-2,3-diphenyl-1*H*-indole	×	×	×
22	21-j	7-(3′-Bromophenylaminomethyl)-4,5,6-trimethoxy-2,3-diphenyl-1*H*-indole	×	×	×
23	21-k	7-(4′-Bromophenylaminomethyl)-4,5,6-trimethoxy-2,3-diphenyl-1*H*-indole	×	×	×
24	21-L	4,5,6-Trimethoxy-7-(3′,4′-dimethylphenylaminomethyl)-2,3-diphenyl-1*H*-indole	×	×	×

n = 5 (five animals were used for each above mentioned activity), ×: no activity, ¥: pro-inflammatory, ¶: pain intensifier (anti-analgesic), Ð: pyretic (increases body temperature), ǂǂ: anti-inflammatory, Aǂǂ: analgesic, Pǂǂ: anti-pyretic.

### Carrageenan induced paw edema in mice

**Figure 1:**
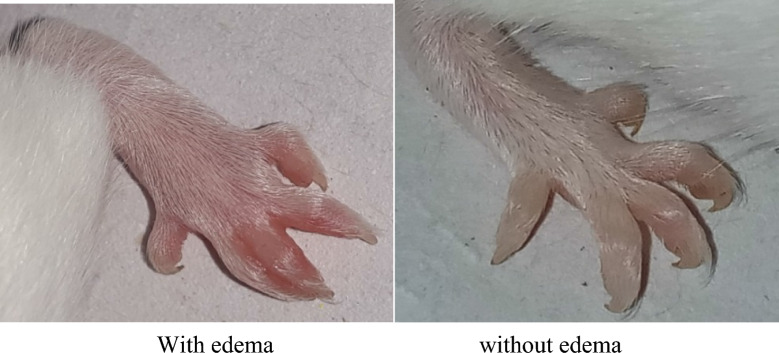
A comparison of mouse paw with and without paw edema.

As shown in the table below (Table 2) administration of 10 mg/kg of both the 3a-II and 4a-II and the indomethacin (10 mg/kg) has prominently reduced paw edema (Fig. 1) with in 60 min after the first injection as compared to the NC group which took 80 min for the paw edema to subside.

**Table 2:** Effect of test chemicals on carrageenan-induced paw edema.

S. no	Chemical code	Effect	Time to subside paw edema
1	Control	Nil	180 ± .57^d^
2	Indomethacin	Anti-inflammatory	90 ± .55^c^
3	3a-ll	Anti-inflammatory	80 ± .56^a^
4	4a-ll	Anti-inflammatory	85 ± .57^b^

Statistical analysis (ANOVA: two factors), group means ± SEM, ^a–d^Anyone two groups not sharing a lower case letters differ significantly from each other.

### Anti-pyretic activity:

The two chemicals (3a-II and 4a-II) have shown antipyretic activity almost parallel to Paracetamol as enumerated in the Table 3.

**Table 3:** Antipyretic activity.

S. no	Groups	Before yeast	After 18 h	Temperature after treatment		
				1 h	2 h	3 h
1	Control	37.88 ± 0.1^a^	38.5 ± 0.1^a^	38.2 ± 0.02^a^	38.4 ± 0.1^b^	38.4 ± 0.03^c^
2	Paracetamol	37.97 ± 0.1^a^	38.6 ± 0.01^a^	38.3 ± 0.1^a^	38.1 ± 0.1^a^	38 ± 0.02^b^
3	3a-II	37.9 ± 0.1^a^	38.5 ± 0.1^a^	38.2 ± 0.1^a^	38.1 ± 0.03^a^	38 ± 0.02^ab^
4	4a-II	37.8 ± 0.4^a^	38.5 ± 0.1^a^	38.3 ± 0.1^a^	38.2 ± 0.02^a^	38.1 ± 0.02^b^

Statistical analysis (ANOVA: two factors), group means ± SEM, ^a–d^Anyone two groups not sharing a lower case letters differ significantly from each other.

### Analgesic activity:

As shown in Table 4 administration of indomethacin (10 mg/kg) has significantly extended the time threshold at 60 and 90 min but after 90 min there is found the same trend of decline in time threshold like all other groups control, 3a-II and 4a-II chemicals at 30, 60, 90,120 and 180 min by comparing them with 0 min (standard timing).

**Table 4:** Analgesic activity.

S. no	Groups	0 min	30 min	60 min	90 min	120 min	180 min
1	Control	5.67 ± 0.57^a^	5.23 ± 0.68^a^	4.74 ± 0.34^a^	4.37 ± 0.47^a^	4.16 ± 0.62^a^	4.02 ± 0.54^a^
2	Indomethacin	5.97 ± 0.84^a^	5.92 ± 0.46^a^	6.44 ± 0.08^a^	6.81 ± 0.75^a^	5.76 ± 0.87^a^	5.59 ± 0.70^a^
3	3a-II	8.33 ± 0.57^a^	8.33 ± 0.57^a^	6.67 ± 0.57^a^	6 ± 0.00^a^	6 ± 0.00^a^	5.67 ± 0.57^a^
4	4a-II	6.33 ± 0.57^a^	6 ± 0.00^a^	6 ± 0.57^a^	5.33 ± 0.57^a^	4.67 ± 0.57^a^	4 ± 0.00^a^

Data is expressed as mean ± SD of n = 5. *P˂0.05 compared with control. *SD* standard deviation.

*0 min: initial response of animals before administration of any treatment (NT) No treatment;*30, 60,…..180 min: response of animal after 30, 60,….180 min of administration of treatment.

Statistical analysis (ANOVA: two factors), group means ± SEM, ^a–d^Anyone two groups not sharing a lower case letters differ significantly from each other.

The results of analgesic activity are shown in following table.

### Gastroprotective activity

#### General observations

**Figure 2:**
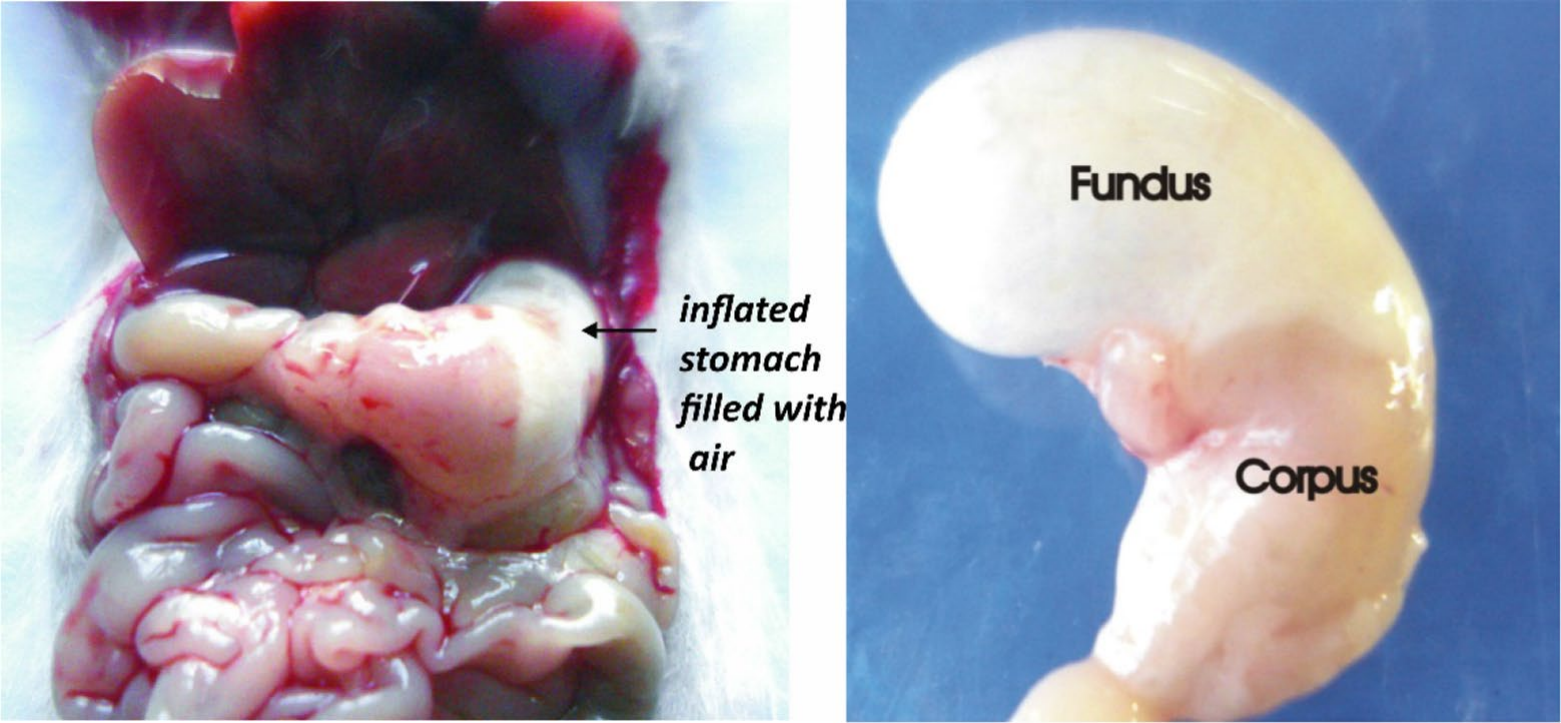
In-situ observation of mouse stomach.

No major morphological changes like architectural derangements, color and size variations (ischemia, redness, swelling, and cirrhosis) were observed in visceral organs in-situ in any of the study groups except swelling in the fundus region of the stomach in PC (indomethacin treated) group (Fig. 2).

#### Anatomy and morphology of the stomach

**Figure 3:**
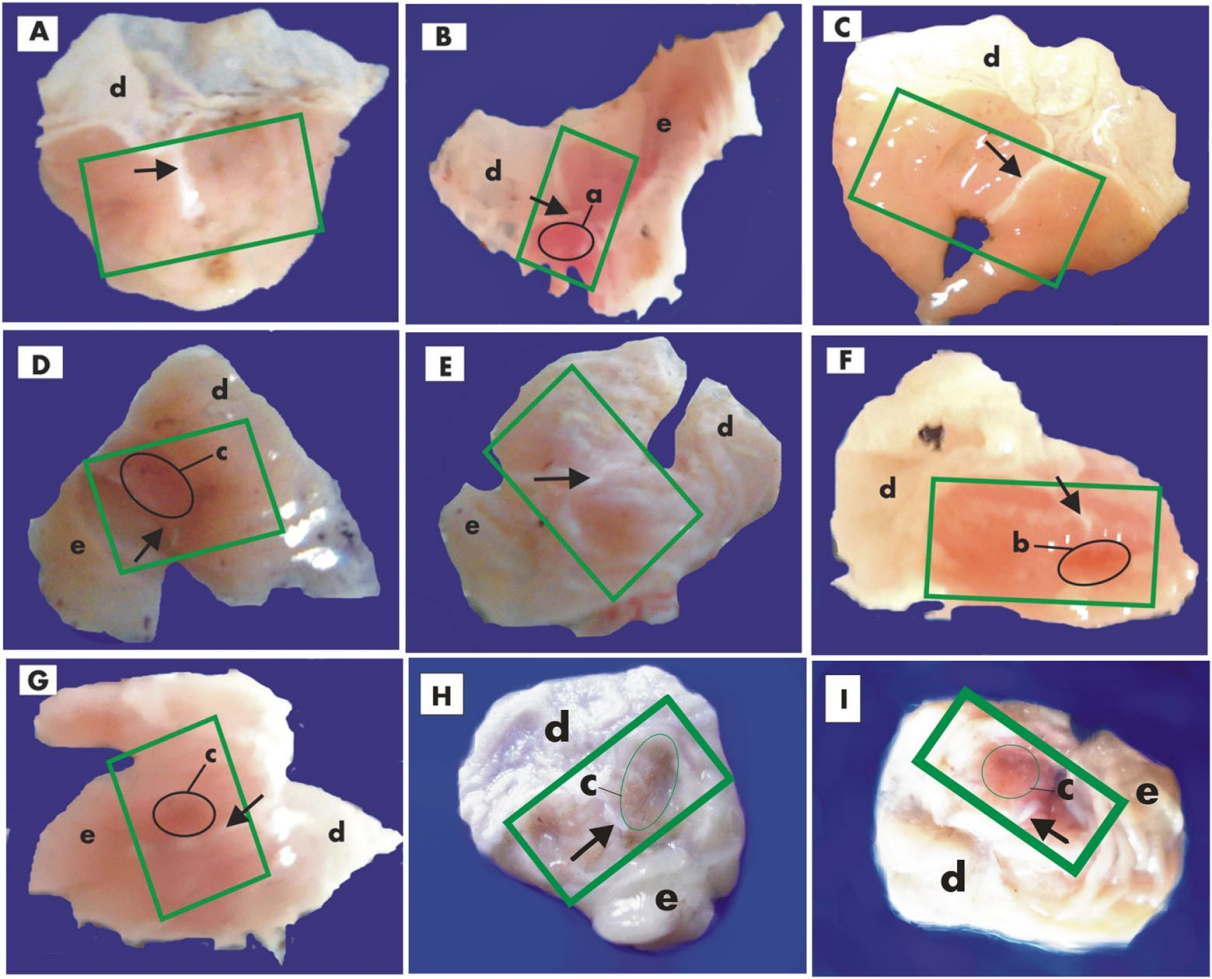
(**A**) Control, (**B**); Indomethacin, (**C**) Omeprazole, (**D**) 3a-20 mg/kg, (**E**) 3a-40mgkg, (**F**) 4a-20 mg/kg, (**G**) 4a-40 mg/kg, (**H**) 3a-4a-II (10 mg/kg), (**I**) 3a-4a-II (20 mg/kg): Arrow in above image showed esophagus, a: highly inflamed stomach wall, b: medium inflammation, c: low inflammation, d: cardiac stomach, e: pyloric stomach, Green square box showed corpus region of stomach (no gastric lesions are visible).

Whereas the NC control group animals have shown no signs of gastric mucosal lesions and /or inflammation (Fig. 3A); The study of the internal lining of the stomach in the PC group (indomethacin fed group) showed that later-minor curvature region was highly prone to mucosal lesions, inflammation, and ulcerations. Thus inflamed mucosal lining along with peptic ulcerations were the common feature of the animals in the indomethacin treated group (Fig. 3B). In the PC-O (Omeprazole treated) group animals the study of the interlining of the stomach showed and very small scattered patches of lesions and inflammation / redness indicating its strong anti-ulcerative properties (Fig. 3C). Peptic mucosal inflammations of indomethacin treatment were also subsided in all four PC-3a-II and 4a-II post-treated groups, however treated group 4a-II (20 mg/kg) had shown very minimal recovery to gastric lesions (Fig. 3F) and the subsidence of gastric lesion or inflammation in PC-3a-II (20 mg/kg) and PC-4a-II (40 mg/kg) were almost similar (Fig. 3D,G), whereas almost complete rescuing cover to the pro-inflammatory effect of indomethacin was observed only in PC-3a-II (40 mg/kg) group (Fig. 3E). In combination dose groups 3a-4a-II (10 and 20 mg/kg) rescuing signs were also observed against indomethacin-induced gastric inflammation (Fig. 3H,I).

### Histological results

**Figure 4:**
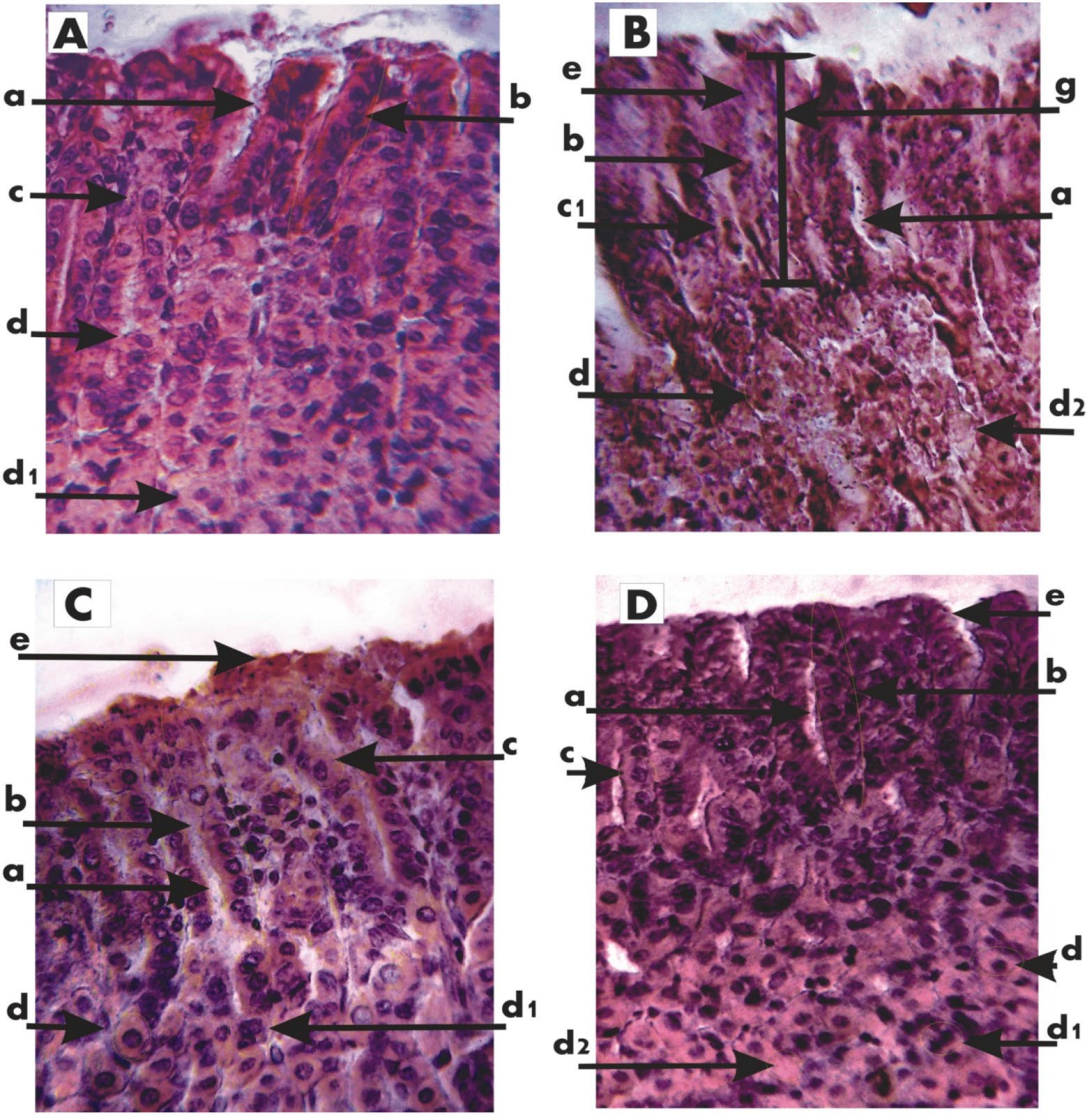

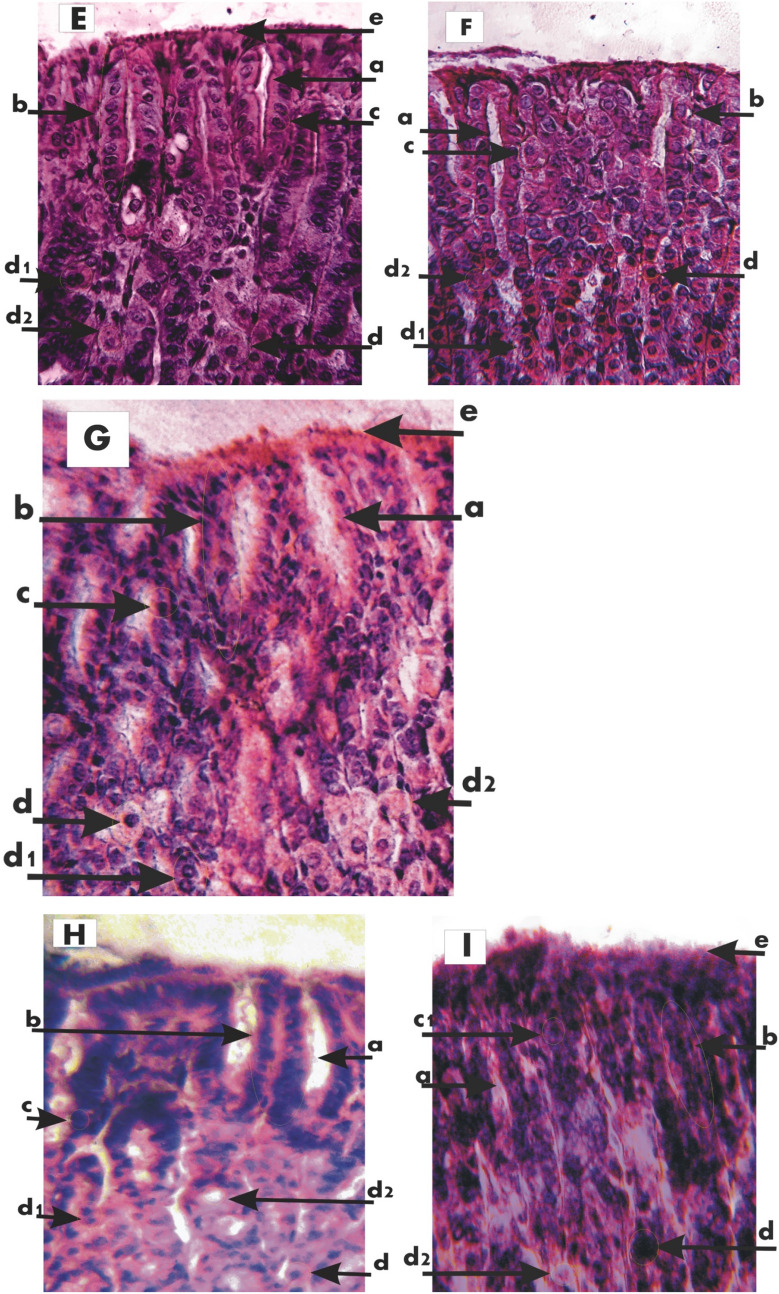
Histological stained section of Mouse stomach (**A**) Control, (**B**) Indomethacin: (a). Pit, (b). Pit strand, (c). Pit cell, (c1).Shrunken Pit cell, (d). Parietal cell, (d1). Mitotic parietal cell, (d2). Apoptotic parietal cell, (e). Mucous, (f). Chief cells, (g). Ulcerated area. (**C**) Omeprazole, (**D**) 3a (20 mg/kg) (a). Pit, (b). Pit strand, (c). Pit cell, (d). Parietal cell, (d1). Mitotic parietal cell, (d2). Apoptotic parietal cell, (e). Mucous, (f). Chief cells. (**E**) 3a (40 mg/kg), (**F**) 4a (20 mg/kg) (a). Pit, (b). Pit strand, (c). Pit cell, (d). Parietal cell, (d1). Mitotic parietal cell, (d2). Apoptotic parietal cell, (e). Mucous, (f). Chief cells. (**G**) 4a (40 mg/kg) (a). Pit, (b). Pit strand, (c). Pit cell, (d). Parietal cell, (d1). Mitotic parietal cell, (d2). Apoptotic parietal cell, (e). Mucous. (**H**) 3a-4a-II (10 mg/kg), (**I**) 3a-4a-II (20 mg/kg) (a). Pit, (b). Pit strand, (c). Pit cell, (d). Parietal cell, (d1). Mitotic parietal cell, (d2). Apoptotic parietal cell, (e). Mucous.

The NC group stomach sections have shown normal gastric infrastructure in terms of gastric pit cells (foveolar columnar cells) covered completely with epithelial layer and proper gastric pits in between gastric pit strands. The middle section has shown frequently occurring acid-secreting parietal cells with normal zymogen cells on the baseline of the section. (Fig. 4A).

In the indomethacin-treated group, there was an almost complete disruption of gastric pits. The pit and parietal cells were rarely observed while the majority were seen in various stages of necrosis (cytoplasmic vacuolations, nuclear disfigurements, etc.), while the epithelial linings were worn-out at various scattered places (Fig. 4B).

A very good histo-architecture of gastric structures like—the evenly distributed gastric pits containing the secretory pit cells and parietal cells were observed in the PC-O group, and a few mucoid secretions were also observed above the epithelial lining (Fig. 4C).

In comparison to the PC group the PC-3a-II (20 mg/kg) group has shown recovery of peptic lesions—as the majority of its parietal cells were seen normal; some functional pits were seen however majority were clogged with mucous (Fig. 4D). As compared to PC-3a-II (20 mg/kg) the in PC-3a-II (40 mg/kg) group showed wider mean pit diameter and the pit cells were also larger in diameter showing stored mucous while epithelial lining was continuous and properly laid out. Some pits also showed filled with mucous secretions. Some aggregation of debris was seen in the parietal cells layer however individual cells of this layer were also seen in various stages of mitosis nevertheless some of the parietal cells were also showing signs of necrosis (Fig. 4E).

The responses to the post 20 and 40 mg/kg of 4a-II treatments to that of the PC indicated pit and partial cell recovery; the pits were deepened almost approaching the parietal cell layer. Mucoid secretions were abundant in the pits. Some stem cells were also seen in the 40 mg/kg post-treatment group (Fig. 4F,G).but in combination groups in which combine a dose of both chemicals was used the 3a-4a-II (10 mg/kg)group showed more rehabilitated effects as compared to 3a-4a-II (20 mg/kg) because in 3a-4a-II (10 mg/kg) group wider gastric pits with less mucoid secretions and increased number of pit cells within pits were seen along with a lot of parietal cells in mitotic phase and in other groups the complete infrastructure of pits was disrupted and covered with mucous. Parietal cells also showed necrosis with wider spaces among them (Fig. 4H,I).

### Micrometric results

The Mean Cross-Sectional Area (CSA) of the parietal / pit cells and the width of the pits have shown significant (*p* ≤ 0.5) variations among the groups (ANOVA). The minimum mean CSA of the parietal cells was observed in 3a-4aII (20 mg/kg), 3a-4aII (10 mg/kg), 4a-II (20 mg/kg), 4a-II (40 mg/kg), control and 3a-II (20 mg/kg) while significantly higher mean CSA was observed in PC-O, 3a-II (40 mg/kg) and PC groups. The minimum mean CSA of pit cells was seen in 3a-4aII (20 mg/kg), 4a-II (20 mg/kg), 3a-4aII (10 mg/kg), 3a-II (40 mg/kg), 3a-II (20 mg/kg), 4a-II (40 mg/kg) and PC (in increasing order) while NC and PC-O showed highest mean CSAs (increasing order) while almost the same trend was found in width of a pit that is narrow in all groups as compared to NC and PC-O groups (Table 5).

**Table 5:** Variations in micrometric parameters of mice stomach.

S. no	Parameters	NC	PC	PC-O	3a-II (20 mg/kg)	3a-II (40 mg/kg)	4a-II (20 mg/kg)	4a-II (40 mg/kg)	3a-4a-II (10 mg/kg)	3a-4a-II (20 mg/kg)
1	CSA of parietal cells (µ^2^)***	0.39 ± 0.27d	0.081 ± 0.15 cd	0.46 ± 0.08abc	0.34 ± 0.08abc	0.53 ± 0.05bc	0.24 ± 0.06a	0.27 ± 0.08ab	0.29 ± .03abc	0.23 ± 0.01a
2	CSA of a pit cell*	0.73 ± 0.08ab	0.51 ± 0.07a	0.92 ± 0.12ab	0.43 ± 0.06a	0.38 ± 0.02a	0.27 ± 0.01a	0.447 ± 1.3b	0.30 ± 0.04a	0.23 ± 0.03a
3	Width of pit***	2.39 ± 0.21b	0.47 ± 0.07ab	3.83 ± 0.30c	1.27 ± 0.22a	1.31 ± 0.12ab	0.76 ± 0.01a	1.76 ± 0.24ab	1.36 ± 0.34ab	1.22 ± 0.29a

Statistical analysis (ANOVA: two factors), *p ≤ 0.05–0.01; **p ≤ 0.001; ***p ≤ .0001, CSA: Cross sectional area, group means ± SEM,^a–d^Anyone two groups not sharing a lower case letters differ significantly from each other.

### Heamatological results

Analysis of data (ANOVA) for different hematological parameters has shown a significant difference among the groups while MCV, MCH, neutrophils, and lymphocyte concentration data showed no significant difference among the groups. Tucky’s multiple range test (TMRT) was used to show variations between the individual groups and has been shown by different lowercase letters with the group mean values in the table below (Table 6).

**Table 6:** Hematological results.

S. no	Parameters	NC	PC	PC-O	3a-II (20 mg/kg)	3a-II (40 mg/kg)	4a-II (20 mg/kg)	4a-II (40 mg/kg)	3a-4a-II (10 mg/kg)	3a-4a-II (20 mg/kg)
1	TLC (× 10^3^/µL)	9.93 ± 2.35a	7.23 ± 2.41a	8 ± 1.21a	5.7 ± 1.97a	5.3 ± 0.4a	7 ± 1.8a	6.7 ± 1.4a	7.24 ± 1.6a	8.42 ± 0.66a
2	RBC (× 10^6^/µL)	6.69 ± 3.53a	7.1 ± 0.4a	6.7 ± 0.77a	8.38 ± 1.36a	6.08 ± 0.97a	6.11 ± 0.76a	8.86 ± 0.94a	7.29 ± 0.61a	8.2 ± 0.45a
3	Hb (g/dl)	9.53 ± 4.65a	11.33 ± 0.65a	11 ± 1.126a	12.17 ± 1.89a	9.55 ± 0.75a	10.63 ± 1.10a	13.43 ± 1.79a	12.97 ± 0.79a	14.07 ± 0.58a
4	HCT (PCV) %	34.1 ± 17.96a	40.53 ± 2.50a	35.87 ± 3.6a	42.06 ± 8.64a	32.75 ± 2.95a	37.25 ± 3.16a	47.57 ± 4.93a	42.7 ± 3.9a	49.8 ± 2.31a
5	MCV (fl)**	50.93 ± 1.10a	56.87 ± 2.08ab	53.36 ± 1.44a	49.9 ± 2.42a	69.45 ± 11.15b	57.71 ± 3.10ab	53.7 ± 1.77a	58.53 ± 1.60a	60.87 ± 1.13a
6	MCH (pg)**	14.67 ± 1.72a	15.9 ± 0.53a	16.37 ± 0.25a	14.53 ± 0.21a	15.77 ± 1.32a	16.85 ± 0.28a	15.13 ± 0.81a	17.87 ± 0.48a	17.2 ± 0.25a
7	MCHC (g/dl)	28.77 ± 2.97a	27.97 ± 0.15a	30.67 ± 0.40a	29.2 ± 1.74a	28.8 ± 0.75a	28.53 ± 0.55a	28.2 ± 0.81a	30.5 ± 1.5a	28.27 ± 0.29a
8	Neutrophils (%)**	8.33 ± 10.21a	35.67 ± 26.76ab	19 ± 18.24a	31 ± 24.75ab	40 ± 30ab	82.67 ± 11.68ab	19 ± 26.8a	33.1 ± 11.9a	31.8 ± 0.95a
9	Lymphocytes (%)**	88.67 ± 11.93b	59.67 ± 30.53ab	76.33 ± 19.39ab	65.67 ± 26.1ab	56.5 ± 31.5aab	15 ± 10a	76.33 ± 32.34ab	66 ± 11.2a	67 ± 1.04a
10	Monocytes (%)	1.67 ± 1.15a	2.67 ± 2.88a	2.67 ± 1.15a	2 ± 1.0a	2 ± 1.0a	2 ± 1.0a	2.67 ± 2.89a	0.30 ± 0.30a	.00 ± 0.00a
11	Eosinophils (%)	1.33 ± 0.57a	2 ± 1.73a	2 ± 0.00a	1.33 ± 0.57a	1.5 ± 0.5a	1.5 ± 0.5a	2 ± 2.65a	.47 ± 0.42a	.17 ± 0.09a

Statistical analysis (ANOVA: two factors), *p ≤ 0.05–0.01; **p ≤ 0.001; ***p ≤ 0.0001, group means ± SEM, ^a–d^Anyone two groups not sharing a lower case letters differ significantly from each other.

## Discussion

Twenty-four novel indole amines were tested for their biological activity. Initially antipyretic, anti-inflammatory, and analgesic activities were performed, and based on results 3a-II and 4a-II were selected for organ pathological profiling as they have shown bio-medicinal properties for all the three initially performed activities (Table 1). Additionally screen out their safety for peptic lesions, ulceration, and gastric bleeding intra-gastric observations were also performed- as the previously existing perception about the NSAIDs (aspirin, indomethacin, and ibuprofen) indicate that these are generally prone to cause these gastric disturbances^19^.

The results have shown that, unlike most the NSAIDs, these two indole amines (3a-II and 4a-II) were not only capable to show analgesic (Table 3), antipyretic (Table 4) and inflammation subsiding capacities (Table 2), but also they harbor good capacity for decreasing induced gastric lesions caused by very high dose (300 mg/kg) of indomethacin treatment.

The analgesic activity is performed by the hot plate method in which pain is induced that generates the reflexes at the spinal level and usually, these pain reflexes are controlled by the brainstem and cortical portion of the brain^20^. In the hot plate method, thermal heat is used to induce pain in the specimen to note the behavior like jumping, withdrawal and licking of paws, etc. This hot plate method is used to induce acute pains by heat-mediated damage of tissues which releases peripheral mediators^16,21^.

In the present study, λ*-*carrageenan is used to perform anti-inflammatory activity. Literature proved that the inflammation caused by *λ*-carrageenan usually occurs both at the cellular and molecular levels. In this mechanism, inflammation occurs in two phases. Like, various inflammatory mediators like serotonin, histamine, and bradykinin are released in the first phase and second phase cytokines as PGs, IL-6, IL-1 *β, and *TNF-*α are generated*^22,23^*. The level of* COX-2 which causes the production of PGs (prostaglandins) in response to inflammation is also shown maximum in the paw edema at the late phase^23^. The probable reason for these two chemicals 3a-II and 4a-II which have shown anti-inflammatory effects is that they may lower the level of inflammatory mediators at the site of inflammation.

Those agents or compounds which have the potential to lower the body temperature are known as antipyretics. Fever is usually caused by some disease, infection, inflammation, or any type of tissue damage^23^ and the body requires a delicate balance of temperature for its regulation for which mainly the hypothalamus helps to regulate the body temperature by maintaining a set point. Exogenously, NSAIDs are important to regulate body temperature or give relief from fever by blocking the COX-2 production of prostaglandins (PGs)^24,25^. In the present study, it has been seen that exposure to 3a-II and 4a-II indoles reduces the body temperature of the specimen which is comparable to the standard drug Paracetamol.

The unique feature of these drugs is their gastroprotective capacity which is unparalleled to any of the existing NSAIDs. As histological and micrometric both results have supported the gastroprotective potential of the above-mentioned chemicals in comparison to NC, PC, and PC-O groups (Fig. 4A–C). Both chemicals at their 40 mg/kg (Fig. 4F,G) concentration showed more prominent and rescuing effects like intact epithelial lining above the gastric pits, continuous structure of pits with wider spaces among them, and most of the parietal cells in different phases of mitosis which is a sign of rehabilitation that cells are going to rescue themselves against necrosis caused by the high dose of indomethacin as compared to 20 mg/kg (4D and 4E) concentration. Due to this rehabilitative potential, these chemicals were also tested in mixture dose as 4a-II chemical showed more regenerative effects and 3a-II have greater rescuing potential that’s why 40 mg/kg of 4a-II chemical was chosen for both groups of combination dose and only concentrations of 3a-II kept vary but the group containing 10 mg/kg 3a-II chemical (Fig. 4H) only showed desired effects rather than 3a-4a-II (20 mg/kg)dose (Fig. 4I). Micrometric results (CSA of parietal cells, CSA of a pit cell and width of pit) and hematological results with *p* ≤ 0.5 also support the histological results (Tables 5 and 6). Additionally, findings have shown that these two novel indole amines are also systemically safe either administered at individual or combination doses. These findings point out their importance as anti-inflammatory, analgesic, and anti-pyretic compounds along with gastroprotective potential. The proposed mechanism of these indoles might be due to the inhibition of PGs by suppressing the level of inflammatory mediators that are the major cause of symptoms of inflammation (swelling, pyrexia, pain, edema accumulation, etc.). So, our findings recommended the use of these indoles for pain relief and fever than NSAIDs because these synthetic compounds not only provide relief from fever and pain but also showed very good gastroprotective agents against ulcers. Further pre-clinical and clinical trials are required before these drugs are moved towards pharmacological registration etc. and also their safety profile for other vital organs is necessary. This study could open new therapeutic potentials for synthetic compounds in the treatment of inflammatory diseases including peptic ulcers.

## Conclusion

Based on current observations, it can be concluded that both chemicals 3a-II and 4a-II play possess significant anti-inflammatory, analgesic, and anti-pyretic potentials along with gastro protective effects against indomethacin-induced gastric ulcer which are almost parallel in action to commercially available anti-ulcergenic drug omeprazole.

## Data Availability

All data generated or analyzed during this study are included in this published article.

## Abbreviations

3a-II: *N*-(4′-Chlorophenyl) (4,6-dimethoxy-2,3-diphenyl-1*H*-indol-7-yl)methanimine
4a-II: *N*-(4′-Methylphenyl) (4,6-dimethoxy-2,3-diphenyl-1*H*-indol-7-yl)methanimine
DMSO: Dimethyl sulfoxide
NSD: Normal saline + 1% DMSO
ANOVA: Analysis of Variance
CSA: Cross sectional area
TLC: Total leukocyte count
RBC: Red blood cells
HCT (PCV): Hematocrit or Packed cell volume
MCHC: Mean corpuscular hemoglobin concentration
MCH: Mean corpuscular hemoglobin
MCV: Mean corpuscular volume
PGs: Prostaglandins
IL-6: Interlukin-6
IL-1: Interlukin-1
TNF-α: Tumor necrosis factor-α
COX-2: Cyclooxygenase-2
