# Author Correction: Evaluation of the anti-inflammatory, analgesic, anti-pyretic and anti-ulcerogenic potentials of synthetic indole derivatives

**DOI:** 10.1038/s41598-024-61715-x

**Published:** 2024-05-14

**Authors:** Saira Siddique, Khawaja Raees Ahmad, Syed Kashif Nawaz, Abdul Rauf Raza, Syeda Nadia Ahmad, Rabiyah Ali, Iram Inayat, Sadia Suleman, Muhammad Ali Kanwal, Muhammad Usman

**Affiliations:** 1 Department of Zoology, University of Sargodha, Sargodha, Punjab Pakistan; 2 Govt. Graduate Ambala Muslim College, Sargodha, Pakistan; 3 Institute of Chemistry, University of Sargodha, Punjab, Pakistan; 4 University of Chakwal, Chakwal, Pakistan

Correction to: *Scientific Reports* 10.1038/s41598-023-35640-4, published online 27 May 2023

Abdul Rauf Raza was omitted from the author list in the original version of this Article.

The Author Contributions section now reads:

“K.R.A. conceived and designed the experiments. S.S., R.A. and M.A.K. performed the experiments. M.U. helped in chemicals and dose preparations. K.R.A., S.S., I.I. and S.N.A. analyzed the data. S.S. and S.K.N. wrote the manuscript. A.R.R. provided the 24 indole derivatives for research work. All authors read and approved the final manuscript.”

In addition, the Acknowledgements section was incomplete.

“We thank the University of Sargodha, Sargodha, Pakistan for permission to conduct this study and provision of appropriate funding. We also thank the lab and animal house facilities for their participation in this research work.”

now reads:

“The authors thank the Higher Education Commission, Government of Pakistan, Islamabad for providing financial support in the form of a research project (HEC-20-3873). We also thank the University of Sargodha, Sargodha (Pakistan) for permission to conduct this study on animal models.”

The original Article has been corrected.

